# Photodynamic therapy effect of *m*-THPC (Foscan®) *in vivo*: correlation with pharmacokinetics

**DOI:** 10.1038/sj.bjc.6601101

**Published:** 2003-07-15

**Authors:** H J Jones, D I Vernon, S B Brown

**Affiliations:** 1 DI Vernon School of Biochemistry and Molecular Biology, Centre for Photobiology and Photodynamic Therapy, University of Leeds, Leeds LS2 9JT, UK

**Keywords:** Foscan, m-THPC, temoporfin, photodynamic therapy, pharmacokinetics

## Abstract

*m*-Tetra(hydroxyphenyl)chlorin (m-THPC, Foscan, Temoporfin) has an unusually high photodynamic efficacy which cannot be explained by its photochemical properties alone. *In vivo* interactions are therefore of critical importance in determining this high potency. The pharmacokinetics of m-THPC in a rat tumour model was determined using ^14^C m-THPC in an LSBD_1_ fibrosarcoma implanted into BDIX rats. The photodynamic therapy (PDT) efficacy was determined at different drug administrations to light intervals and correlated with the tumour and plasma pharmacokinetic data. The plasma pharmacokinetics of m-THPC can be interpreted by compartmental analysis as having three half-lives of 0.46, 6.91 and 82.5 h, with a small initial volume of distribution, suggesting retention in the vascular compartment. Tissues of the reticuloendothelial system showed high accumulation of m-THPC, particularly the liver. PDT efficacy of m-THPC over the same time course seemed to exhibit two peaks of activity (2 and 24 h), in terms of tumour growth delay with the peak at 24 h postinjection correlating to the maximum tumour concentration. Investigation on tumour cells isolated from m-THPC-treated tumours suggested that the peak PDT activity at 2 h represents an effect on the vasculature while the peak at 24 h shows a more direct response. These results indicate that the *in vivo* PDT effect of m-THPC occurs via several mechanisms.

Photodynamic therapy (PDT) is becoming accepted as an alternative to conventional cancer treatments for certain indications and other nononcological conditions. It is based on a tumour accumulation of a photosensitiser, which, when activated by light, results in tumour destruction via reactive oxygen species (Ochsner, 1997). The selectivity of PDT relies upon the targeting of the light delivery in combination with the accumulation/retention of the photosensitiser in malignant tissue. The time interval between photosensitiser administration and light delivery is crucial for the optimal clinical efficacy of PDT. The distribution of the photosensitiser both in the tumour as a whole and throughout the tumour compartments is dependent on this interval as well as *in vivo* interactions that affect photosensitiser aggregation, delivery and uptake (Boyle and Dolphin, 1996).

*m*-Tetra (hydroxyphenyl)chlorin (m-THPC), a second-generation photosensitiser, has already been shown to be more potent than Photofrin (PII). It has been suggested that it is up to 200 times more powerful (Van Geel *et al*, 1995; Ball *et al*, 1999). This figure takes into account the drug dose required (0.15 mg kg^−1^ compared to 10 mg kg^−1^ for PII) and the lower light doses necessary (30 J cm^−1^ rather than 150 J cm^−1^) to produce similar PDT results. However, the reason for this effectiveness remains unresolved even considering the advantageous photoproperties of increased molar absorption coefficient and a favourable shift in the wavelength maximum (652 nm rather than 630 nm) (Bonnett *et al*, 1989).

Preclinical studies have already shown the importance of the drug to light interval in m-THPC PDT (Ris *et al*, 1991). The longer time intervals are preferable to optimise PDT treatment by limiting normal tissue damage. Another consideration with m-THPC is the unusual human plasma pharmacokinetics. After an intravenous injection, the plasma level initially falls exponentially but at approximately 10 h it begins to rise to a secondary peak at 24 h (Ronn *et al*, 1996; Glanzmann *et al*, 1998). This may change the level of m-THPC in the tumour vasculature and the rate of uptake of m-THPC in the tumour giving different PDT responses. In addition, m-THPC initially forms an aggregated complex in human plasma that slowly redistributes to the lipoproteins where it is predominantly monomeric. These different aggregation states of the photosensitiser would alter its tumour uptake and localisation (Hopkinson *et al*, 1999).

To help unravel the *in vivo* interactions of m-THPC, the PDT effect needs to be correlated with drug level and tumour localisation. In this study, we have used a ^14^C radiolabelled preparation of m-THPC to investigate the pharmacokinetics of the sensitiser in male BDIX rats implanted with a subcutaneous LSBD_1_ fibrosarcoma. The same time course was then used to assess PDT efficacy using tumour growth delay as the end point. The main aim of this study was to correlate tumour and tissue concentrations with PDT response.

To study the mechanism of action of m-THPC, the effects of irradiation on tumour cells isolated from m-THPC-containing tumours were compared to the effects of the tumour *in vivo*.

## MATERIALS AND METHODS

*m*-Tetra (hydroxyphenyl)chlorin and ^14^C m-THPC were supplied by Scotia QuantaNova. The sensitiser was dissolved as recommended for intravenous injection in a mixture of polyethylene glycol 400 : ethanol : water (3 : 2 : 5 by volume). Radioactive sensitiser was diluted with unlabelled m-THPC to a specific activity of 16.9 mCi mmol^−1^ and a final concentration of 0.6 mg ml^−1^. Unlabelled m-THPC was also prepared as a 0.6 mg ml^−1^ solution and the solutions were stored in 0.5 ml aliquots at −20°C.

### Animal and tumour model

Animal experiments were performed under protocols approved by the local experimental animal welfare committee and conformed to National and European regulations for animal experimentation. Experiments were covered by Home Office premises, project and personal licenses as well as keeping within the UKCCCR guidelines on use of animals for experimental neoplasia (Workman *et al*, 1998).

Male BDIX rats were used at 12–13 weeks old and weighed approximately 300–400 g. The tumour model used was a poorly differentiated fibrosarcoma (LSBD_1_), which grows as a sphere causing no skin ulceration and no attachment to the muscle below. The tumour was implanted as a subcutaneous fragment (1 mm) when the rats weighed 300–400 g (Gilson *et al*, 1990).

### Tumour measurement and administration of m-THPC

Rats were weighed and tumours measured daily. Measurements were taken on three perpendicular axes using callipers to calculate the mean tumour diameter. When tumours had reached a mean diameter of 8–10 mm, approximately 10 days after implantation, they were suitable for PDT treatment and were entered into the study. *m*-Tetra (hydroxyphenyl)chlorin (0.3 mg kg^−1^) was injected intravenously via the penile vein.

^14^C m-THPC or unlabelled m-THPC was administered at 0, 2, 4, 6, 18, 24, 48, 72 or 96 h before the tumour was predicted to reach 8–10 mm in diameter. This was determined from the known tumour growth kinetics. In addition, a 168-h time point was used in the pharmacokinetic study that had a slightly different protocol, consisting of m-THPC administration when the tumour was 8–10 mm. All procedures were carried out using UKCCCR guidelines (Workman *et al*, 1998).

### Pharmacokinetic study

After the predetermined times, the animals were killed by deep anaesthesia with ether, followed by cervical dislocation. The tissues selected for dissection were tumour, liver, heart, kidney, lung, muscle (skeletal muscle of the hind leg) and skin (right abdominal wall). Dissection was carried out under subdued light, and all tissues were washed in isotonic saline and blotted dry. Triplicate samples of each tissue (90–110 mg) were weighed directly into scintillation vials. Blood samples (2.5 ml) were collected by heart puncture (immediately after cervical dislocation). The samples were then diluted with an equal volume of heparin solution (10 U ml^−1^) and centrifuged to separate the plasma. Three animals were used per time point and all samples were stored at −20°C. Procedures were carried out using UKCCCR guidelines (Workman *et al*, 1998).

Each tissue sample was digested by addition of 1 ml of 1 M sodium hydroxide to the scintillation vial and shaking overnight in the dark at 40°C. Scintillation fluid (10 ml) (Ultima Gold, Perkin Elmer Life Sciences, Cambridge, U.K.) were added to each vial followed by vigorous shaking. Radioactivity was determined using a Packard Liquid Scintillation Analyzer (1900TR). An internal radiolabelled standard, ^14^C *n*-hexadecane was used to determine the counting efficiency. The plasma samples were analysed using 200 *μ*l aliquots (in triplicate for each animal) mixed with 10 ml scintillation fluid and counted as above.

For each animal, the mean tissue concentration was calculated. The mean and the standard deviation (s.d.) for all three animals at each time point were calculated. The plasma pharmacokinetics was analysed by compartmental and noncompartmental mathematical methods (Yamaoka *et al*, 1978; Clark and Smith, 1986).

### Photodynamic therapy on the LSBD_1_ tumour

Tumours were irradiated interstitially using a 200 *μ*m optical fibre with a 0.5 cm diffusing tip (Feather *et al*, 1989). A light dose of 50 J was delivered at 100 mW from a 652 nm diode laser (Diomed Inc., Boston, USA). During treatment, rats were placed on a heated mat and kept anaesthetised using a halothane inhalation system.

Tumour growth delays were calculated by subtracting the time taken for control animals to reach a mean tumour diameter of 15 mm, from the time taken for treated tumours to reach 15 mm. This end point was used to assess the response to the PDT treatment and used for comparison between time points (Thomlinson and Craddock, 1967).

### Photodynamic therapy on isolated LSBD_1_ tumour cells

The LSBD_1_ tumour was implanted subcutaneously in the BDIX male rat. When the tumours reached a diameter of 8–10 mm, m-THPC was administered intravenously at a dose of 0.3 mg kg^−1^ (0.6 mg ml^−1^ solution) and the animals were killed at 2 and 24 h together with a control animal. Procedures were carried out using UKCCCR guidelines (Workman *et al*, 1998). The tumour was excised (intact) under aseptic conditions and subdued lighting and was cut into quarters on a sterile Petri dish. The white tumour tissue was removed from the outer tumour capsule. The tumour was shredded using two scalpels and incubated with 10 ml of 0.25% collagenase II solution containing 1% BSA (added just before incubation) for 1–2 h at 37°C, shaking intermittently.

The tumour cell suspension was passed through a 150 *μ*m nylon filter mesh and the cells collected by centrifugation (MSE Mistral 2000, 500***g***, 5 min). The collagenase II mixture was poured off and the remaining cells resuspended in 10 ml of phosphate-buffered saline (PBS). The cell suspension was then passed through a 100 *μ*m nylon filter mesh and centrifuged as above. The cells were again resuspended in 10 ml of PBS and centrifuged. This was repeated until the suspension was clear, then the cells were suspended in 5 ml Dubecco's modified Eagle's medium (DMEM) containing 10% FCS and 1% glutamine. The concentration of cells in the suspension was determined using a haemocytometer. Cells were seeded at 1 × 10^5^ per well of a 96-well plate, in 200 *μ*l of medium, and left for 4 h to allow cell attachment. Immediately before illumination, the medium was removed from the cells and replaced with PBS.

The plates were irradiated using a xenon arc lamp fitted with a 652 nm filter (±15 nm). The cells were treated with light doses of 0, 2.5, 5, 10, 20 and 40 J cm^−2^, by varying the illumination time (0–16 min) and then incubated for 18–24 h at 37°C before determining cell survival by the MTT assay (Mosmann, 1983).

### Statistical analysis

Statistical analysis was carried out using analysis of variance in the computer program SAS 6.12 system for Windows™ (SAS Institute Inc., SAS Campus Drive, NC, USA).

## RESULTS

### Plasma pharmacokinetics of m-THPC in the BDIX rat

Figure 1

**Figure 1:**
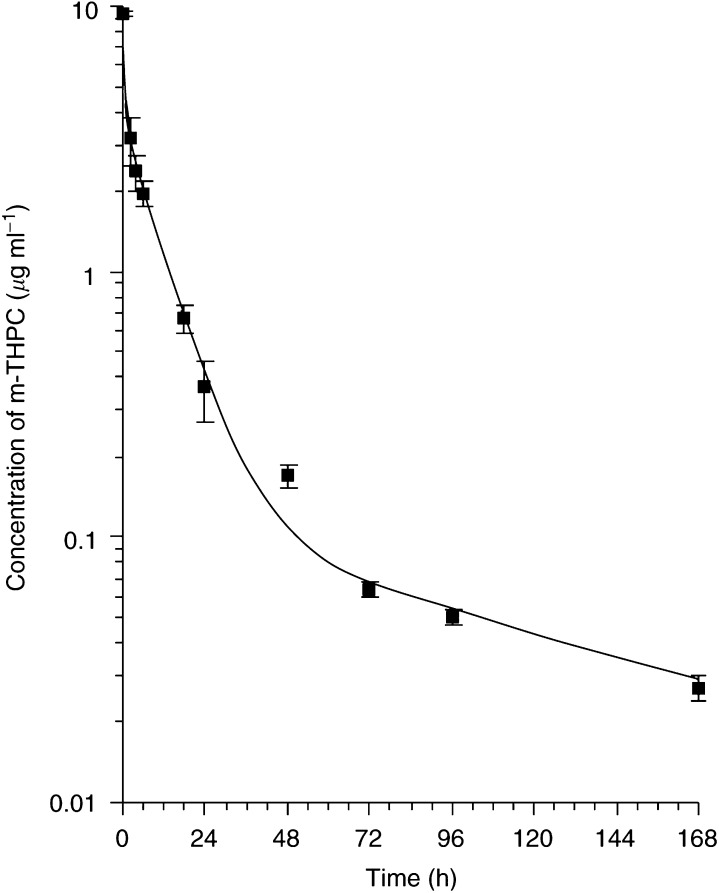
Semilog plot of the pharmacokinetics of ^14^C m-THPC in the plasma of the BDIX rat injected with 0.3 mg kg^−1^ (data points show the mean±s.d., *n*=3).

shows the levels of m-THPC in rat plasma at various times after administration (0. 3 mg kg^−1^). The plasma levels were initially high (9.37 *μ*g ml^−1^) and appear to declined exponentially as would normally be expected for an intravenous injection. The clearance from plasma, however, did not follow a simple monoexponential decay but could be fitted to multiple exponentials, and because of this the data were analysed by both a noncompartmental and a compartmental approach (Yamaoko *et al*, 1978; Clark and Smith, 1986). With a compartmental approach, the data best fit a three exponential decay described by the equation *C*=5.89 e^−1.51t^+3.36 e^−0.1t^+0.120 e^−0.0084t^. This model gives three half-lives of 0.46, 6.91 and 82.5 h and elimination rate constants of 1.51, 0.1 and 0.0084 h^−1^, respectively (Table 1A

**Table 1:** Pharmacokinetic parameters calculated using (A) the exponential equations of the three compartment model equation of 5.89 e^−1.51t^+3.36 e^−0.1t^+0.120 e^−0.0084t^ (A e^−*α*t^+Be^−*β*t^+Ce^−*γ*t^), and (B) the noncompartmental method (dose=0.3 mg kg^−1^ m-THPC)

**Pharmacokinetic parameter**	**Result**
(A)	
Initial concentration (*C*_0_)	9.37 *μ*g ml^−1^
Initial volume of distribution (*V*_d_)	32.0 ml kg^−1^
*V*_d_ of first compartment	50.9 ml kg^−1^
*V*_d_ of second compartment	89.3 ml kg^−1^
*V*_d_ of third compartment	2500 ml kg^−1^
*t*_*1/2*_ of first compartment	0.46 h
*t*_*1/2*_ of second compartment	6.91 h
*t*_*1/2*_ of third compartment	82.5 h

(B)	
Mean residence time (MRT)	32.5 h
Elimination constant (*K*_el_)	0.0307 h^−1^
Half-life (*t*_*1/2*_)	22.6 h
Plasma clearance	5.29 ml kg^−1^ h^−1^
Volume of distribution (*V*_d_)	172 ml kg^−1^

).

A noncompartmental analysis using statistical moments gives a better overall view of the pharmacokinetics as it compensates for individual data being collected from a number of animals.

For the noncompartmental approach, the calculation method was as follows.

1. The plasma concentration of m-THPC at time zero (*C*_0_) was estimated by extrapolation of the log concentration *vs* time graph back to zero.
2. The slope of the log linear (elimination) phase (*k*′_∞_) was estimated between 72 and 168 h.
3. The area under the concentration *vs* time graph (AUC) was calculated from time zero to the last time point (*T*(last)) by the trapezoidal rule and from *T*(last) to infinity as Area=*C*(last)/*k*′, where *C*(last) is the plasma concentration at the last time point (168 h).
4. The area under the (concentration × time) *vs* time curve (moment curve, AUMC) was calculated from time zero to *T*(last) by the trapezoidal rule and *T*(last) to infinity as Area=*C*(last)/(*k*′)^2^.
5. Plasma clearance *C*_L_=DOSE/AUC.
6. Mean residence time MRT=AUMC/AUC.
7. Volume of distribution *V*_d_=C_L_ × MRT.
8. Half-life *T*_1/2_=MRT × ln(2).
9. Elimination rate constant *K*_el_ =1/MRT.

Table 1B shows the noncompartmental pharmacokinetic parameters obtained. This approach gives an elimination rate constant of 0.03 h^−1^ and a biological half-life of 22.6 h.

From the compartmental analysis, the initial volume of distribution of the central compartment, calculated from the initial plasma concentration (Dose/*C*_0_), was estimated to be 32.0 ml kg^−1^. The phases of the three compartments, however, have volumes of distribution of 50.9, 89.3 and 2500 ml kg^−1^ (Table 1A) while noncompartmental analysis shows an average *V*_d_ of 172 ml kg^−1^. These values suggest that some tissues preferentially accumulate m-THPC (i.e. the volumes are so much larger than the blood volume).

### Tissue distribution of m-THPC in the BDIX rat

**Figure 2:**
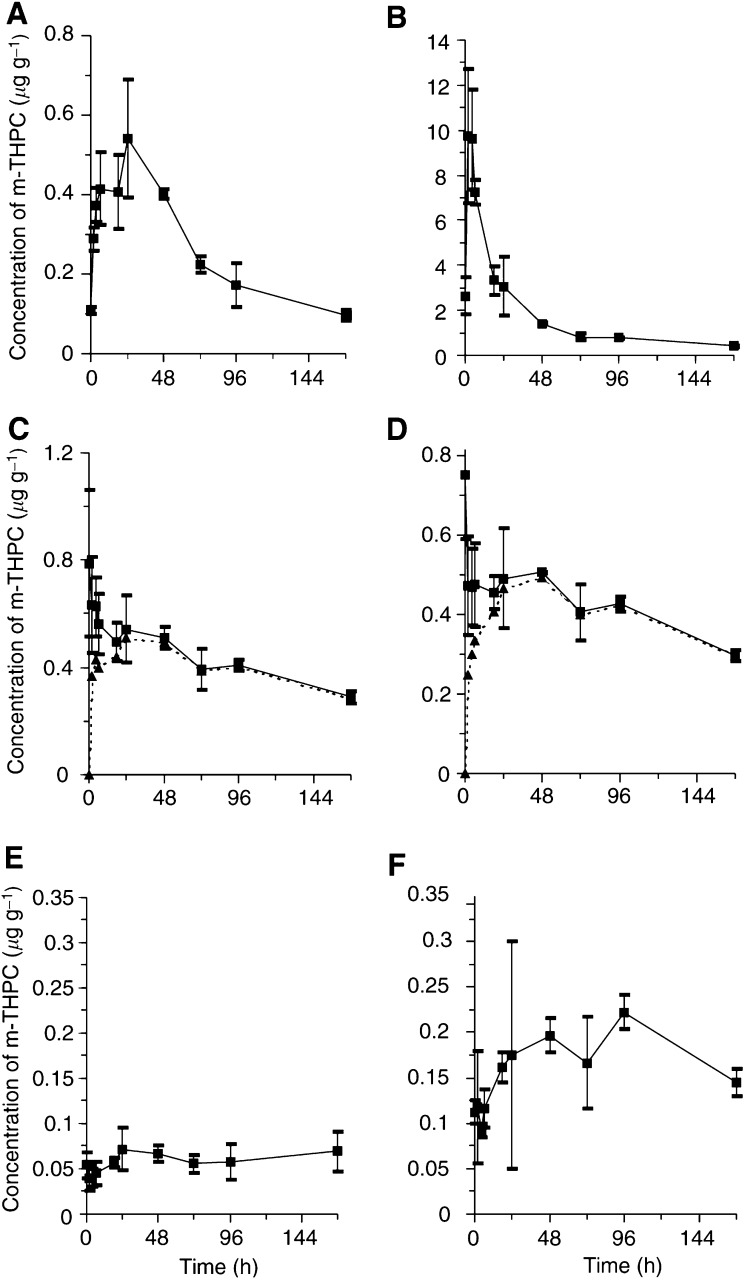
Concentration of m-THPC in selected tissues of the BDIX rat at different times after an intravenous injection of ^14^C m-THPC (0.3 mg kg^−1^). Data corrected for blood content of the tissue (–––). (**A**) LSBD_1_ tumour, (**B**) liver, (**C**) kidney, (**D**) heart, (**E**) muscle and (**F**) skin. (data points show the mean±s.d., *n*=3).

All tissues studied showed an accumulation of m-THPC with the highly perfused tissues such as liver and kidney accumulating higher levels of m-THPC than the peripheral tissues like skin and muscle (Figure 2

). The contribution of the blood levels of m-THPC to the levels in the different tissues can be estimated if it is assumed that the initial tissue value (<2 min) was due solely to the blood content and this contribution decreased in the same manner as the plasma pharmacokinetics. Therefore, the m-THPC levels are also shown after subtraction of the m-THPC present in the tissue due its blood content. This mainly affected the tissues with high blood volume and which showed peak concentrations at the first time point (<2 min) (Table 2

**Table 2:** Time and concentration of maximal levels of ^14^C m-THPC in each tissue of the BDIX rat (data represent mean±s.d. (*n*=3))

**Tissue**	**Peak (h)**	**Concentration (*μ*g g^−1^)**
Tumour	24	0.54±0.15
Liver	2	9.74±3.01
Skin	96	0.22±0.02
Muscle	24	0.07±0.02
Plasma	<5 min	9.37±0.19

).

Tumour concentration rises quickly in the first 24 h with the concentration remaining above 0.4 *μ*g g^−1^ from 6 to 48 h (Figure 2A). The maximum level in the tumour is 0.54±0.15 *μ*g g^−1^ at 24 h and after 48 h the concentration in the tumour declines quickly. The liver has an extremely high initial concentration of m-THPC and it accumulates approximately 30 times the original dose of 0.3 mg kg^−1^. This suggests some type of significant uptake mechanism for m-THPC consistent with its role as a detoxification organ. The kidney and heart have similar levels of m-THPC, approximately twice the original dose. The sensitiser accumulates rapidly in the first 2 h slowing to a peak at 24–48 h similar to the pattern of uptake by the liver. The muscle and skin accumulate the least amount of m-THPC.

The elimination rates from the tissues (Table 3

**Table 3:** Elimination rate constants and half-lives of the terminal phase of elimination in several tissues of the BDIX rat injected with 0.3 mg kg^−1^^14^C m-THPC

**Tissue**	**Elimination rate constant (× 10^3^ h^−1^)**	**Half-life (h)**
Plasma	8.40	82.5
Tumour	8.46	81.9
Liver	7.13	97.3
Kidney	3.52	196.7
Heart	3.61	191.9

) were calculated from the log concentration *vs* time graphs using the last three data points. These were very similar in some tissues, suggesting they are part of one compartment for elimination. The terminal elimination rates of the tumour and liver are similar to that of the plasma, indicating that m-THPC diffusing out of these tissues in the later stages of the experiment was immediately eliminated. The kidney and the heart had a similar elimination profile of m-THPC but with a longer half-life, suggesting another possible compartment in the model (Table 3). The muscle and skin showed comparable shaped kinetic profiles, although the m-THPC levels in the muscle were half that of the skin. Once the m-THPC had entered these tissues it did not enter an elimination phase, as the log linear portion of the log concentration *vs* time plot is almost constant from 72–168 h (data not shown). The slow elimination is probably due to slow diffusion out of these tissues. This indicates another possibly longer half-life for m-THPC, which was not encountered in the plasma pharmacokinetics during this time course.

A high tumour to normal tissue ratio for a photosensitiser is particularly important in PDT to ensure maximum effect in the tumour with minimal normal tissue damage. There was no general trend for the tissues although some tissues can be grouped together with the highly perfused tissues, such as the liver, having low ratios over the entire time course (Table 4

**Table 4:** Ratio of ^14^C m-THPC concentration in the LSBD_1_ tumour compared to normal tissue (dose: 0.3 mg kg^−1^^14^C m-THPC)

**Time (h)**	**Muscle**	**Skin**	**Liver**
2	7.40	2.44	0.03
4	9.39	4.06	0.04
6	10.36	2.43	0.05
24	7.57	3.09	0.18
72	4.04	1.35	0.28
96	2.97	0.77	0.22

Concentrations used for calculations were not corrected for blood content of the tissue.

). Tumour to tissue ratios are high for muscle and skin peaking at 10.4 and 4.1, respectively. These ratios occur at earlier time points than peak tumour concentrations (24 h) although the ratio remains high (i.e. above 1) for most of the time course.

### Photodynamic therapy efficacy of m-THPC in the BDIX rat

**Figure 3:**
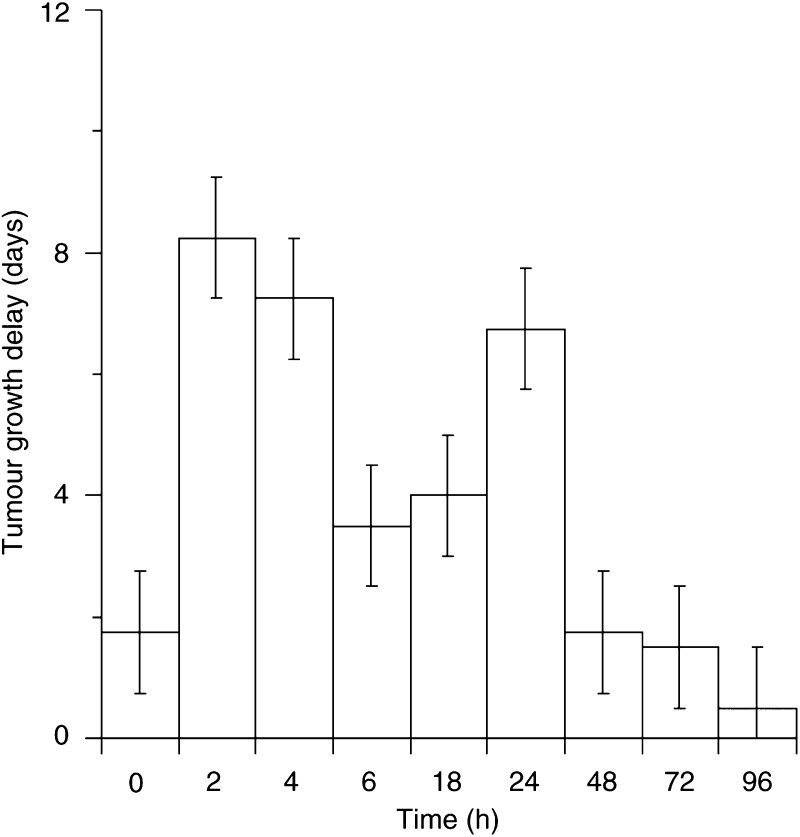
Tumour growth delay of the LSBD_1_ tumour following interstitial treatment at different times after intravenous injection of 0.3 mg kg^−1^ m-THPC in the BDIX rat. A light dose of 50 J (100 mW) was delivered via a 200 *μ*m optical fibre with a 0.5 cm diffusing tip. (data points show the mean±s.d., *n*⩾3).

Tumour growth delay of treated tumours was calculated by comparison with the rate of growth of tumours in control animals. Light only and drug only controls showed no deviation from control animals. The pattern of tumour growth delay over the time course was similar for both a 652 nm diode laser (Figure 3

) and a 652 nm Copper Vapour pumped dye laser (data not shown) with no significant difference between the two sources. After most treatments, there was an immediate increase in tumour diameter that regressed after approximately 24 h. This oedema is a short-lived inflammatory response due more to the insertion of the fibre than PDT and has also been observed with other photosensitisers in this model (Cruse-Sawyer *et al*, 1998).

Photodynamic therapy efficacy peaked at 2–4 h postadministration with an apparent second peak at 24 h. There was no significant difference between treatments at 0, 6, 48, 72 and 96 h for the diode laser (*P*>0.05). However, the 2 h interval was significantly different to all time points except 4 and 24 h (*P*<0.05). Hence, there were two peaks in tumour growth delay, an early peak around 2–4 h followed by a second peak around 24 h. At the time intervals that caused significant tumour growth delay, there were periods of tumour stasis that lasted up to 5 days after the initial regression; this was then followed by a period of increased growth at a slower rate than the controls (data not shown).

### Photodynamic therapy treatment of isolated LSDB_1_ tumour cells

**Figure 4:**
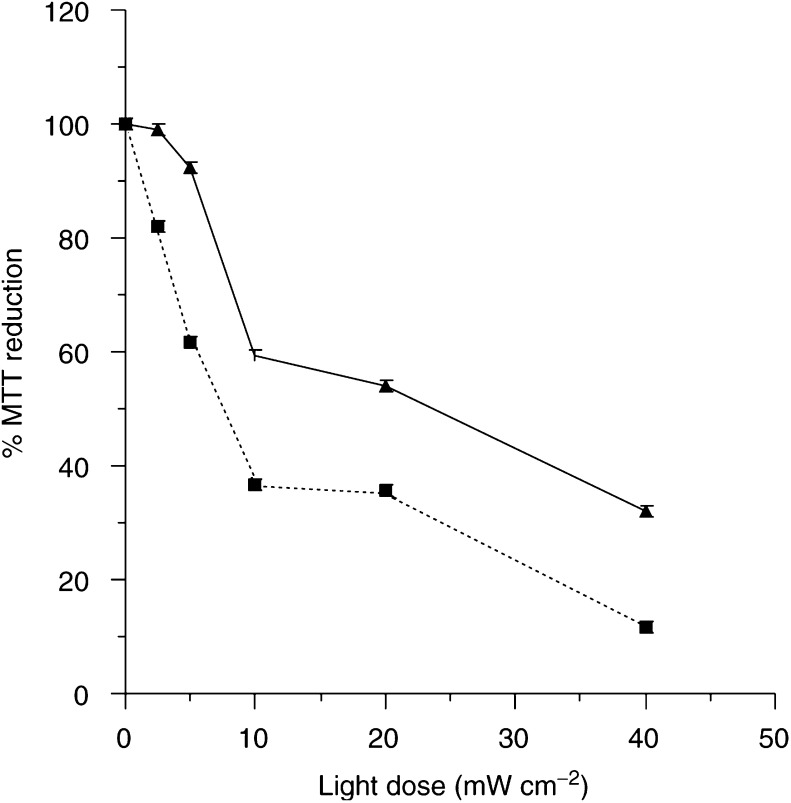
Phototoxicity of cells isolated from a LSBD_1_ tumour after intravenous injection of m-THPC (0.3 mg kg^−1^) followed by *in vitro* light treatment with xenon arc lamp (5–40 mW cm^−2^). Cells isolated after 2 h (—) and 24 h (–––) (data points show the mean±s.d., *n*=3).

The LSBD_1_ tumours were removed 2 and 24 h after injection of 0.3 mg kg^−1^ m-THPC. The tumour cells were isolated, and treated *in vitro* to investigate the extent of direct cell kill compared to indirect cell damage caused by vascular or stromal elements. The amount of m-THPC present in the cell should not change during the 4 h of cell attachment (after isolation), as m-THPC is known to remain in cells even if serum is present extracellularly (Ball *et al*, 1999). Figure 4

shows that the PDT effect is greater in the tumour cells extracted 24 h after injection than those extracted after 2 h. The phototoxicity was dependent on light dose, the higher light doses caused more cell damage in both experiments. At every light dose, cell survival was significantly lower for the cells extracted after 24 h (*P*<0.05).

## DISCUSSION

For any single PDT treatment, there are a variety of important factors that need to be optimised. These range from the choice of photosensitiser and administration dose, to the time interval before treatment, as well as considering any one of the light dose parameters. Preclinical results indicate that the efficacy of m-THPC is related to both the drug dose and the interval between administration and illumination (Ris *et al*, 1993a, 1993b). However, the efficacy is also complicated by many *in vivo* interactions, such as aggregation and protein binding (Glanzmann *et al*, 1998; Hopkinson *et al*, 1999). This study aims to correlate m-THPC pharmacokinetics with PDT efficacy and the mechanism of PDT damage. Radiolabelled (^14^C) m-THPC was used in this study to avoid the problems encountered with noninvasive induced fluorescence determination (Lin, 1990).

The plasma pharmacokinetics of m-THPC in BDIX rats best fitted three exponential decays and considering the influence of each component a general prediction can be made. The three phases of elimination represent a rapid distribution phase, a second phase that consists of mainly elimination with slight redistribution, while the final phase shows elimination from other tissues. There is also a high initial retention in the vasculature that is confirmed by initial volume of the distribution (32.0 ml kg^−1^), which is small compared to the blood volume of a rat (50–70 ml kg^−1^). *m*-Tetra (hydrophenyl)chlorin may be initially retained in the vascular system, possibly due to protein binding or precipitation (Clark and Smith, 1986). This is consistent with plasma binding studies with m-THPC that shows an unusual initial protein-binding pattern for a hydrophobic photosensitiser (Hopkinson *et al*, 1999).

A similar study using ^14^C-labelled m-THPC in mice (Cramers *et al*, 2003) has shown that the decrease in photosensitiser levels in plasma can be described by a second-order decay with half-lives of 1.3 and 20.9 h. Although these half-lives cannot be directly compared to the three compartment analysis for the rat data, they do show a similar rapid initial distribution phase and a much longer elimination phase. In the Syrian hamster, a much longer plasma half-life (2–3 days) has been demonstrated. This is similar to the terminal half-life of 82.5 h in the BDIX rat. It must be pointed out however that the early plasma pharmacokinetic data is likely to be a reflection of the plasma lipoprotein profile of the particular species being studied. Rats and mice have very little low-density lipoprotein (LDL), whereas the profile of the hamster is similar to that of humans although the composition of the individual lipoproteins differs slightly (Goulinet and Chapman, 1993).

The present study was too short to show the terminal elimination half-life from the peripheral tissues as determined by the log concentration plots for skin and muscle. However, Whelpton *et al* (1995) showed a terminal half-life of approximately 10 days in mice as measured by excretion rates.

Using a noncompartmental analysis, the biological half-life of m-THPC in the BDIX rat is 22.6 h. This compares with 80 h for a Photofrin equivalent in the same model (Vernon *et al*, 1995). This shows that the clearance of m-THPC is faster than that of Photofrin. Similar pharmacokinetic analyses in humans have determined half-lives as 452 h for Photofrin (Brown *et al*, 1992) and 30 h for m-THPC (Glanzmann *et al*, 1998).

The plasma pharmacokinetics of m-THPC in humans is unusual for an intravenous dose, and several studies show a secondary peak at 24 h (Ronn *et al*, 1996; Glanzmann *et al*, 1998). Ronn *et al* (1996) showed a secondary peak at 4–6 h in rabbit plasma. Although, there is no secondary peak shown in the rat data presented here, the levels at 2 h are still high and it is possible that this phenomenon is much shorter in a rat, hence a peak might be present between 0 and 2 h in this study. There is some evidence from the density gradient ultracentrifugation analysis that m-THPC is initially highly aggregated in rat plasma but disaggregates and redistributes to lipoproteins with time (Hopkinson *et al*, 1999).

High levels of m-THPC in the tumour would suggest a good PDT response, although the intracellular localisation of the photosensitiser is critically important. The tumour concentration rises quickly in the first 6 h with its concentration staying above 0.4 *μ*g g^−1^ from 6 to 48 h. Low tumour concentration at the later time points (48–168 h) could be due to the concentration of m-THPC being diluted due to the rapid growth of the tumour. The LSBD_1_ tumour in this model has a doubling time of approximately 1.5–2 days. These kinetic data are similar to those seen in other tumours and animal models. Whelpton *et al* (1995) showed that tumour concentrations of m-THPC in a mouse model peaked at 24 h at 1.49±0.25 *μ*g g^−1^ and Peng *et al* (1995) demonstrated, in another mouse tumour, a peak at 48 h of approximately 2 *μ*g g^−1^. Cramers *et al* (2003) found similar levels in a human mesothelioma xenograft in BALBlc mice.

The level of m-THPC in other tissues is also important in PDT to predict the possible extent of normal tissue damage. In this study the liver has an extremely high concentration of m-THPC, reflecting its role in the elimination of m-THPC (Alian *et al*, 1994; Whelpton *et al*, 1996). The m-THPC initially disappears rapidly from the liver unlike Photofrin or our own equivalent (PHP), which in the same animal and tumour model stays at a high concentration for several days (Stribbling, 1991). The much slower elimination phase of m-THPC (Table 3) is similar to that of plasma. Hence the photosensitisers have different mechanisms of sequestration or redistribution and removal from the liver, possibly conferring an advantage for m-THPC. One postulation is that m-THPC could disaggregate and redistribute to lipoproteins in the liver before further distribution to other tissues. The kidney, heart and lung all have a relatively high concentration of m-THPC. In the case of lung tissue, the peak concentration of m-THPC reached a maximum of 2 *μ*g g^−1^ and the elimination profile was almost identical to that of the tumour (data not shown). This follows observations that tissues of the reticuloendothelium system accumulate high concentrations of photosensitisers (Gomer and Dougherty, 1979; Alian *et al*, 1994; Peng *et al*, 1995). The skin and muscle accumulated m-THPC more slowly and appear to peak at 24 h and 96 h, respectively (Figure 2). The elimination phase did not occur in the time course of this study and a half-life could not be determined. The levels in the skin remained at peak concentrations throughout the remaining time of the study. This is in agreement with studies in the mouse (Cramers *et al*, 2003). This m-THPC could give rise to prolonged skin sensitivity although it may be rapidly photobleached, due to the sharp absorption bands, avoiding the photosensitivity (Ma *et al*, 1994). Studies in the hamster (Blant *et al*, 2002) have shown that levels of m-THPC in striated muscle are very low, increasing slightly over 200 h. This was shown to be quite different from smooth muscle, which accumulated relatively higher levels and peaked at about 36 h. The data for striated muscle in the BDIX rat presented here are consistent with the data for the hamster model.

PDT efficacy was also determined over the same drug light intervals for direct correlation with tumour sensitiser levels. The major feature of the PDT efficacy time course (drug to light time interval) was that there were two peaks of tumour growth delay (2 and 24 h). In studies in human mesothelioma xenografts, a similar early peak of PDT effect was seen at 3 h mice (Cramers *et al*, 2003). No second peak in efficacy could be seen in this study although the presence of one could not be ruled out. One contributing factor to this difference may be related to the doubling time of the tumours. The LSBD_1_ tumour has a doubling time of about 2 days, whereas that of the mesothelioma xenograft was 15 days (Cramers *et al*, 2003); a greater rate of cell division giving rise to a faster cellular uptake.

The pattern of efficacy does not follow the pattern of m-THPC concentration in the tumour. Significantly different drug concentrations in the tumour (0.29±0.03 *μ*g g^−1^ at 2 h and 0.54±0.15 *μ*g g^−1^ at 24 h) produce similar tumour growth delay (Figure 3). Similarly, PDT efficacy does not parallel the m-THPC concentration in the plasma as has been shown for BPDMA (Lin *et al*, 1998) and m-THPC in the mouse (Cramers *et al*, 2003). This would not explain the peak of activity at 24 h as plasma levels of m-THPC are much lower at 24 h than at 2 h. Finally, a specific localisation has been postulated as a prerequisite for optimum photosensitisation, suggesting that photosensitisers may have different PDT mechanisms at different time points (Star *et al*, 1986; Henderson and Bellnier, 1989).

The mechanisms involved in the initiation of tumour destruction by PDT are difficult to distinguish and are thought to be dependent on the localisation of the photosensitiser due to the small diffusion distance of the reactive oxygen species (Peng *et al*, 1996). Intracellularly localised photosensitisers produce more direct cell kill in comparison to those photosensitisers accumulating in the interstitial space, which cause more vascular and stromal effects. The mode of action of m-THPC in the LSBD_1_ tumour at 2 and 24 h was examined by assessing tumour cell survival after administration of m-THPC *in vivo*, followed by cell isolation and light exposure *in vitro*.

The cells extracted 24 h after injection of m-THPC showed a significantly higher PDT effect than those extracted after 2 h, whereas *in vivo* the effects are relatively similar, indicating either more m-THPC present in the cell or a specific localisation within the cell. Localisation studies of m-THPC have shown strong fluorescence in the vasculature walls at 1 h, while at 24 h m-THPC is still present in the vasculature but also localised intracellularly (Peng *et al*, 1995, 1996). Similar vascular deposition has been determined in the Syrian Hamster (Blant *et al*, 2002). This suggests that the effect at 2 h, probably caused by m-THPC present in the plasma or interstitial space, is due more to damage of the vasculature leading to deprivation of oxygen and nutrients causing tumour cell death and loss of tumour structure (Denekamp, 1992). Menezes Da Silva and Newman (1995) reported that the vascular effect of m-THPC was unlikely to be a direct effect on the vasculature but accumulation at alternative sites that have an indirect effect on blood capillaries. However, at 24 h m-THPC has accumulated intracellularly and has a more direct effect.

## CONCLUSION

The biological half-life of m-THPC in the BDIX rat model, as determined using a noncompartmental pharmacokinetic approach, is 22.6 h. This is much lower than that for a Photofrin equivalent that has a half-life of 80 h in the same model. The photodynamic effect of m-THPC in the BDIX rat fibrosarcoma shows two peaks of activity in the 96 h study. *Ex-vivo* investigations suggest that the early effect at 2 h is probably due to a vascular mechanism possibly linked to high plasma concentrations, with a more direct cell kill mechanism being involved at 24 h. This latter peak correlated with maximum tumour concentrations of m-THPC.
